# Predicting the Potential Suitable Distribution of *Albizia odoratissima* (L. f.) Benth. Under Climate Change Based on the Biomod2 Model

**DOI:** 10.3390/biology14020180

**Published:** 2025-02-10

**Authors:** Zhiting Li, Qiaomiao Ji, Yong Yang, Yunfei Gao, Meng Xu, Yali Guan

**Affiliations:** 1 Ministry of Education Key Laboratory for Ecology of Tropical Islands, Key Laboratory of Tropical Animal and Plant Ecology of Hainan Province, College of Life Sciences, Hainan Normal University, Haikou 571158, China; 920501@hainnu.edu.cn (Z.L.); 202312071300017@hainnu.edu.cn (Q.J.); yybiology@163.com (Y.Y.); 920043@hainnu.edu.cn (Y.G.); 2 Co-Innovation Center for Sustainable Forestry in Southern China, College of Forestry, Nanjing Forestry University, Nanjing 210037, China; xum@njfu.edu.cn

**Keywords:** *Albizia odoratissima*, Biomod2, climate change, potential geographic distributions

## Abstract

*Albizia odoratissima* (L. f.) Benth. is a highly valuable tree species, both economically and ecologically. It has great potential for development. To conserve, develop, and use its genetic resources sustainably, it is crucial to evaluate the potential habitat of *A. odoratissima* and identify the key environmental factors that affect its distribution. In this study, the Biomod2 model was used to predict the potential distribution of *A. odoratissima* in China, using 65 occurrence points and 11 environmental variables for both current and future climate scenarios. The distribution of *A. odoratissima* is mainly influenced by three temperature-related factors: bio4, bio8, and bio11. Our prediction shows that the suitable habitats for *A. odoratissima* are mostly located in several southern provinces of China, totaling 136.98 × 10^4^ km^2^. Under different climate scenarios, the suitable habitat area is expected to shift northward in the future, although the highly suitable areas will remain relatively stable. These findings have important implications for the conservation, development, and sustainable utilization of *A. odoratissima* resources.

## Simple Summary

*Albizia odoratissima* holds significant economic and ecological value, with considerable potential for development. This study used the Biomod2 model to evaluate the impact of climate change on the distribution of *A. odoratissima* in China. The results reveal that the primary factors influencing the distribution of *A. odoratissima* are the Mean Temperature of Coldest Quarter, Temperature Seasonality, and Mean Temperature of Wettest Quarter. Currently, the area of suitable habitat for *A. odoratissima* in China is 136.98 × 10^4^ km^2^. With climate change, the distribution of *A. odoratissima* will gradually expand, and the distribution center will move westward. These findings are crucial for the protection and utilization of *A. odoratissima* resources.

## 1. Introduction

Climate plays a crucial role in shaping the distribution ranges of plant species, and climate change will cause rapid shifts in the distribution of plants [1,2,3]. Since the Industrial Revolution, global temperatures have been steadily rising. Compared to 1880, the current temperature of the Earth has risen by one degree Celsius [4]. Studies indicate that intensified climate change drives habitat loss and fragmentation, threatening species survival and potentially leading to the extinction or decline of endangered species [5,6].

Species distribution models (SDMs) are widely used tools for predicting the potential response of species to climate change [7]. These models can combine environmental variables and known distribution points to assess the ecological requirements of species and project the calculated results in a specific spatiotemporal context to predict the potential distribution status of species [8]. Numerous models, such as BioClim [9], Genetic Algorithm for Rule-set Prediction (GARP) [10], Generalized Linear Models (GLMs), Random Forest (RF), and maximum entropy models (MaxEnt) [11], have been developed to predict species distribution areas [12,13]. Differences in underlying algorithms give each model distinct advantages and limitations, resulting in variable outcomes when applied to the same species. To address this, ensemble models have been developed to improve prediction accuracy by combining multiple models. Biomod2, an R-based platform, integrates several commonly used species distribution models, enhancing predictive precision [14]. To date, the Biomod2 model has been successfully applied to predict the potential distribution areas of various plants, such as *Eucommia ulmoides*, *Luculia pinceana*, and *Salvia hydrangea* [15,16,17].

*Albizia odoratissima* (L. f.) Benth. is an evergreen tall tree species belonging to the Leguminosae family, native to southern China, as well as India, Bangladesh, and Sri Lanka [18]. *A. odoratissima* is characterized by a straight trunk, rapid growth, and high economic value. It has excellent wood properties, significant differences between the heartwood and sapwood, is not easy to crack or deform, and is insect- and corrosion-resistant. These attributes make it a valuable material for the production of musical instruments, furniture, and other high-demand goods [18]. Additionally, extracts from the flowers, leaves, and bark of *A. odoratissima* exhibit potent antibacterial and antioxidant properties, contributing to its significant medicinal value [19,20,21]. Furthermore, *A. odoratissima* belongs to the Caesalpinioideae subfamily of the legume family (The Legume Phylogeny Working Group, LPWG, 2017) [22], which has strong nitrogen fixation ability and is highly resistant to harsh environments, making it an excellent choice for afforestation and soil and water conservation and has important ecological value. Therefore, understanding the distribution patterns and trends of *A. odoratissima* habitats under varying climate scenarios is of great significance for the future introduction, domestication, and management of the species, as well as for the development of effective germplasm resource management strategies.

*A. odoratissima* holds significant development potential. While previous studies primarily focused on its genetic diversity [23], drought resistance mechanism [24] and medicinal value [21], research on its potential suitable habitats remains limited. In this paper, the suitable habitats of *A. odoratissima* were simulated, and the changes in potential suitable areas of *A. odoratissima* under various shared socioeconomic pathways (SSPs) in the future were predicted. The results will provide a theoretical foundation and key insights for the conservation, scientific introduction, and cultivation of *A. odoratissima*.

## 2. Materials and Methods

### 2.1. Data Collection

A total of 324 distribution data for *A. odoratissima* were obtained from published literature and the Global Biodiversity Information Facility (https://www.gbif.org/ (accessed on 20 September 2024)). To mitigate the risk of model over fitting due to over-density of local distribution points, the software ENMTools 1.1.0 [25] was employed to remove duplicate records, retaining only one occurrence point per 20 km × 20 km grid cell. Ultimately, 65 unique distribution records of *A. odoratissima* in China were selected for further analysis (Table S1).

### 2.2. Environmental Variables

In this study, 19 bioclimatic variables with 2.5 min spatial resolution for the historical (1970–2000s) were downloaded from the WorldClim database (http://worldclim.org (accessed on 20 September 2024)). Slope, aspect, and altitude data were extracted from digital elevation model (DEM) data with an accuracy of 500 m, obtained from the Geospatial Data Cloud (http://www.gscloud.cn/ (accessed on 20 September 2024)). To assess the impact of future climate change on species distribution, BCC-CSM2-MR from the CMIP6 were applied, which included four SSPs (SSP126, SSP245, SSP370, and SSP585). Compared with other global circulation models, BCC-CSM2-MR is more suitable for simulating the suitable habitats of species in China [26,27]. All environmental variables in raster format were aligned with the geographic space using raster (https://github.com/rspatial/raster (accessed on 25 September 2024)).

ENMTools was used to conduct correlation analysis on 21 environmental variables. If the correlation coefficient between two variables was high (|r| ≥ 0.8), the variable with the higher contribution to the MaxEnt model, based on trial runs, was retained. Finally, 11 environmental variables were retained for further analysis (Table S2). The contribution of each variable was assessed using logistic regression within the MaxEnt model. The MaxEnt model were optimized by ENMeval [28] with the regularization multiplier (RM) set within the range of 0.5 to 4, with an increase of 0.5 per run, and five different feature combinations (FCs) were tested, namely L, LQ, LQH, LQHP, and LQHPT. Then, the above 40 parameters were combined into ENMeval package 2.04 for verification, and the parameter combination with the lowest delta AICc will be used for model prediction [29].

### 2.3. Model Calibration

In the present study, Biomod2 v4.2-5-2 was used to conduct SDM for *A. odoratissima* [30]. Twelve different algorithms within Biomod2 were employed for model predictions, including artificial neural networks (ANN), flexible discriminant analysis (FDA), classification tree analysis (CTA), generalized additive models (GAMs), generalized boosting models (GBM), generalized linear models (GLM), maximum entropy (MAXENT), maximum entropy new implementation (MAXNET), multivariate adaptive regression splines (MARS), eXtreme gradient boosting (XGBOOST), a random forest (RF), and a species range envelope (SRE). A total of 1000 pseudo-absence points were randomly generated using the random function of the dismo [31]. For each modeling, 75% of the distribution data were randomly selected as the training dataset and remaining used for testing, repeating 10 times. Model performance was evaluated using two metrics: the receiver operating characteristic (ROC) and the True Skill Statistic (TSS) to evaluate model performance. After the evaluation of single models, Biomod2 was used to combine those with TSS values surpassing 0.75 into a final ensemble model.

ArcGIS [32] was used to process the prediction results. The natural breakpoint method was applied to categorize the results into four suitability classes for *A. odoratissima*: unsuitable, low-suitable, medium-suitable, and high-suitable, with the following thresholds: 0–134, 134–448, 448–791, and 791–1000, respectively. To examine the changes in suitable habitat, the SDM toolbox was employed to analyze the geometric centers of suitable areas. By monitoring the shifts in the position of the geometric center, the trend of changes in the suitable habitat of *A. odoratissima* under different climatic conditions were investigated.

## 3. Results

### 3.1. Model Validation

**Figure 1:**
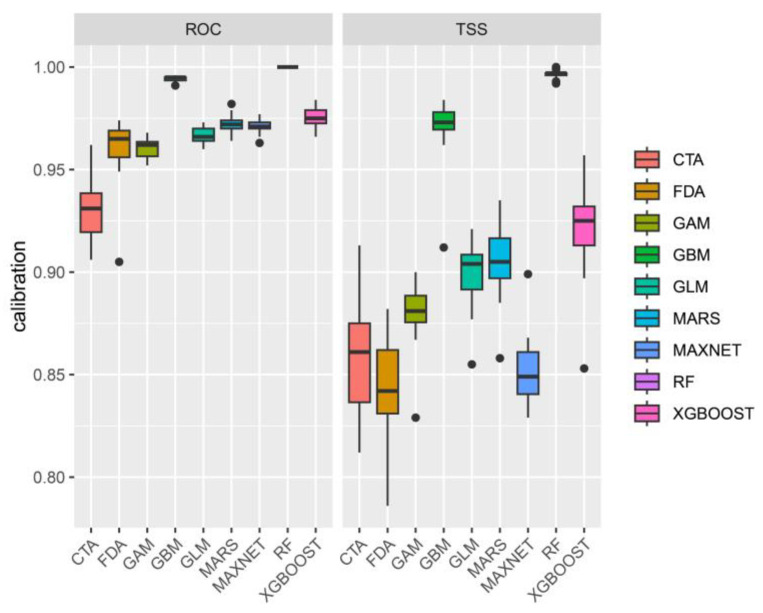
TSS and ROC evaluations of each single-model.

After 10 repeated runs, nine models showed high performance, with TSS values greater than 0.75 and AUC values greater than 0.9 (Figure 1). Among these, the EMca version of the ensemble model exhibited the highest accuracy, with a TSS value of 0.897 and an ROC value of 0.979 (Table 1). Given its outstanding performance, the EMca was selected for further analysis.

### 3.2. Selection of Environmental Variables

**Figure 2:**
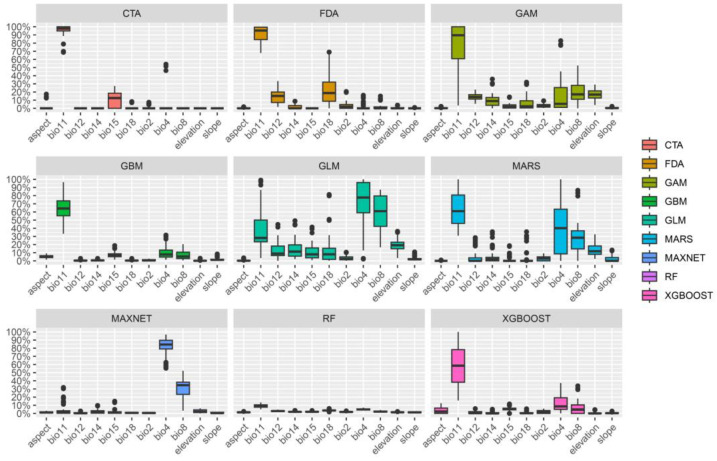
Contribution rate of environmental factors for each single model.

**Figure 3:**
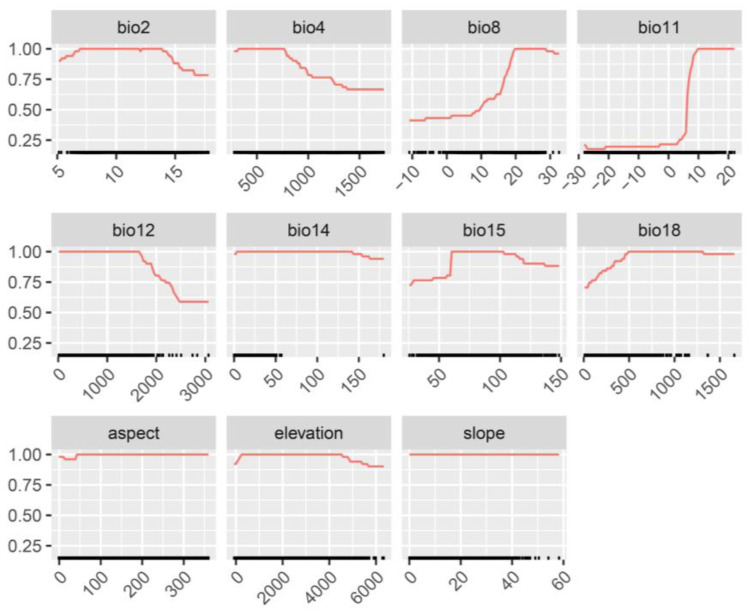
Response curves of *A. odoratissima*.

Based on the results of the MaxEnt model trial run, a total of 11 environmental variables were selected to predict the distribution habitats of *A. odoratissima*. The prediction results from the nine individual models indicate that Mean Temperature of Coldest Quarter (bio11) has the greatest impact on the distribution of *A. odoratissima*, followed by Temperature Seasonality (bio4) and Mean Temperature of Wettest Quarter (bio8) (Figure 2). According to the response curve, *A. odoratissima* exhibits the highest adaptability when the bio11 exceeds 6.28 °C, the bio4 (standard deviation × 100) is below 788.82, and the bio8 is above 18.66 °C (Figure 3). This indicates that *A. odoratissima* is a typical tropical plant, suitable for tropical and south subtropical zone with high temperatures and minimal annual temperature fluctuations.

### 3.3. Current Potential Geographical Distribution in China

**Figure 4:**
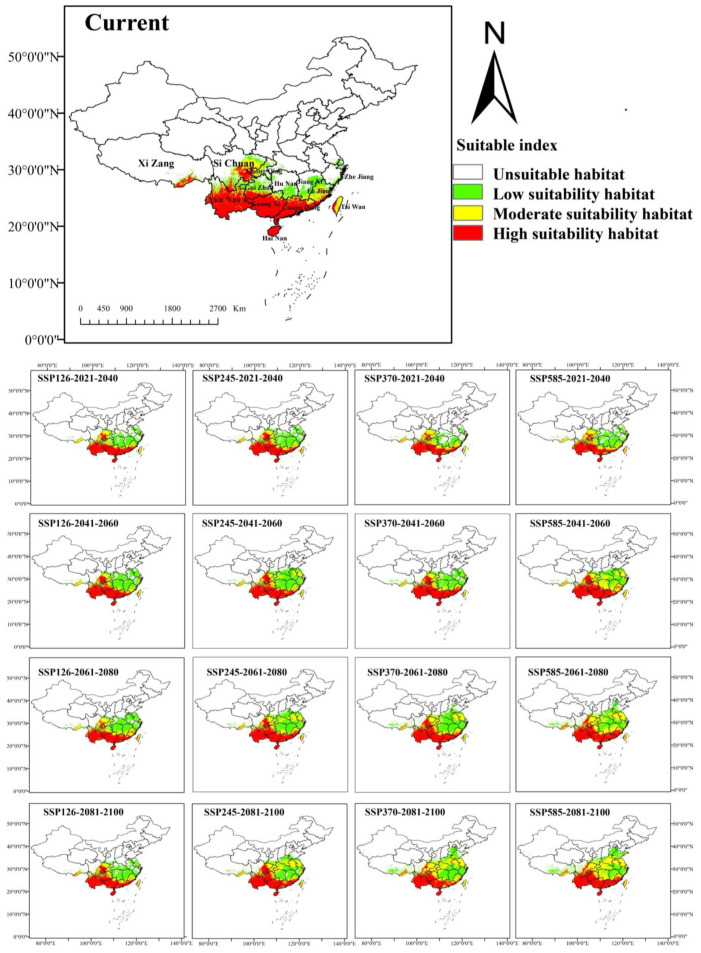
Potential geographic distribution of *A. odoratissima* in China under current and future climatic scenarios.

The potential distribution of *A. odoratissima* in China under both current and future climatic scenarios was predicted using ensemble model community averaging (EMca) (Figure 4). Under the current scenario, the area of highly suitable habitat for *A. odoratissima* covers 75.58 × 10^4^ km^2^, while the moderately suitable habitat area is 28.40 × 10^4^ km^2^, and the low suitable habitat extends over 33.00 × 10^4^ km^2^, totaling 136.98 × 10^4^ km^2^ of suitable habitat. The highly suitable habitats are mainly located in southern China, including the provinces of Hainan, Guangdong, Guangxi, and Yunnan, as well as sporadic areas in Taiwan, Guizhou, Sichuan, Fujian, and Xinjiang. The predicted distribution largely aligns with the actual geographic distribution data for *A. odoratissima*.

### 3.4. Distribution Changes Under Future Climate Conditions

**Figure 5:**
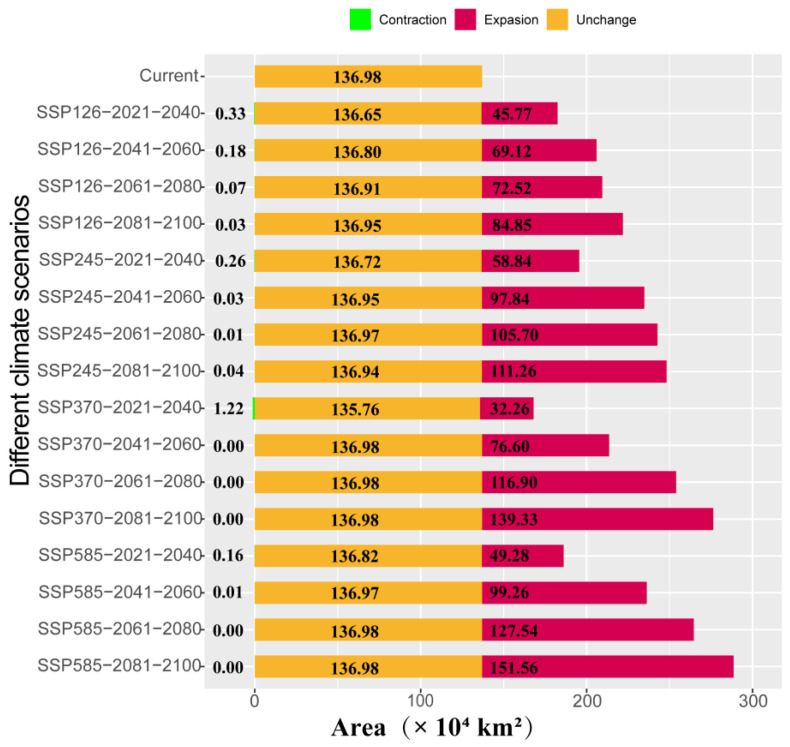
Areal change in *A. odoratissima* habitat in different periods.

Under future climate scenarios, the potential distribution area of *A. odoratissima* in China is expected to gradually expand (Figure 4 and Figure 5). Under four different scenarios, there are significant differences in the size of area expansion. The SSP126 scenario predicts the smallest expansion, with the suitable habitat area increasing to 221.80 × 10^4^ km^2^ between 2081 and 2100, representing a 1.6-fold increase. In contrast, the SSP585 scenario forecasts the largest expansion, with the suitable habitat area growing to 288.54 × 10^4^ km^2^ during the same period, more than doubling in size. The areas of expansion are primarily concentrated in the provinces of Jiangsu, Anhui, Hunan, and Hubei. Notably, there was no significant reduction in habitat under different scenarios (Figure 5).

**Figure 6:**
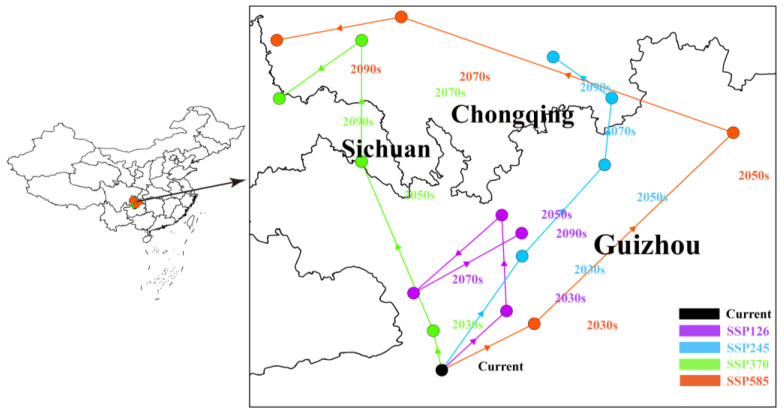
Migration of the center of suitable habitat for *A. odoratissima* under different climatic scenarios. Arrows indicate the direction of migration direction of the center.

The geometric center of potential habitats was used to represent the overall spatial location of potential suitable habitats for *A. odoratissima*. Currently, the geometric center of its potential suitable habitats is located in Guizhou Province (106.517° E, 27.706° N). Under the SSP126 climate scenario, during the 2081–2100 period, the center will shift northward to the northern part of Guizhou Province (106.876° E, 28.324° N), at a distance of 77.23 km from the current distribution centroid. Under the SPS585 climate scenario, the centroid will move northward to Chongqing (105.774° E, 29.193° N) during 2061–2080, at a distance of 180.59 km from its current location (Figure 6).

## 4. Discussion

### 4.1. Evaluation of Modeling Performance

The choice of model is crucial to the accuracy of prediction results. Differences in algorithms, spatial distribution, and sample size among various models can significantly impact both accuracy and predictive performance [33]. Currently, there is no model that can perform well in all prediction tasks [34]. The ensemble modeling approach, which combines the outputs of multiple individual models, provides an effective solution to this challenge. In recent years, ensemble models have gained popularity and are widely used for species distribution predictions, such as for *Centaurea solistialis* [35], moso bamboo [36], and *Fritillaria* [37]. In this study, the TSS value of the EMca ensemble model was 0.897, and the ROC value was 0.979, indicating strong performance and high predictive accuracy.

### 4.2. Critical Environmental Factors Influencing A. odoratissima Distribution

Climate factors exert a significant influence on species distribution patterns, with temperature and precipitation being key determinants [38,39]. The results of this study indicate that the primary climatic factors affecting the distribution of *A. odoratissima* are bio4, bio8, and bio11, all of which are temperature related factors (Table S2). Specifically, the higher the temperature and the smaller the seasonal temperature variation, the greater the adaptability of *A. odoratissima*, which aligns with the climate characteristics of its current distribution areas. It is worth noting that although the high suitability habitats are mainly located in tropical regions with a tropical monsoon climate, high temperatures and humidity, and abundant summer precipitation, the influence of precipitation on the species’ adaptability is relatively low. In fact, as an excellent pioneer species, *A. odoratissima* is drought-resistant and barren [24], with strong nitrogen fixation ability, and has an improving effect on the soil ecological environment. Consequently, precipitation has a relatively minor impact on the adaptability of *A. odoratissima*.

### 4.3. Impact of Climatic Changes on Suitable Habitats for A. odoratissima

In this study, most of the highly suitable habitats for *A. odoratissima* were located south of latitude 25′ N, encompassing the provinces of Hainan, Guangdong, Guangxi, and Yunnan, as well as the southern regions of Fujian, Guizhou, Xinjiang, and Taiwan. Additionally, a significant suitable habitat was identified near latitude 30′ N, primarily within the Sichuan Basin. The Sichuan Basin, surrounded by mountains, experiences milder winter temperatures compared to other regions at the same latitude. This is due to the Daba Mountains to the north, which block the southward movement of cold air, resulting in a more temperate climate [40]. These climatic conditions create a more favorable environment for the growth of *A. odoratissima*.

Global climate change has driven the northward migration of habitats for various species [39,41,42]. Similarly, climate change has caused the centroid of *A. odoratissima*’s distribution to shift northward. The extent of this northward expansion varies under different climatic scenarios. By 2100, under the SSP585 scenario, the distribution area of *A. odoratissima* will reach its maximum, expanding to 288.55 × 10^4^ km^2^, more than doubling the current area. However, the expansion primarily occurs in low and moderately suitable habitats, while the range of highly suitable habitats remains largely unchanged. In addition, with climate change, the predicted distribution area of *A. odoratissima* shows almost no contraction under different SSP scenarios, suggesting that its potential distribution remains relatively stable despite ongoing climate change.

## 5. Conclusions

In this study, ensemble models were employed to investigate the potential geographic distribution of *A. odoratissima* and its changes under different climate change scenarios. The analysis identified that the key factors limiting the distribution of *A. odoratissima* are bio11, bio4, and bio8. Under the current climate, the potential distribution area of *A. odoratissima* is concentrated in southern China, with a total area of 136.98 × 10^4^ km^2^. Highly suitable habitats are mainly located in the provinces of Hainan, Guangdong, Guangxi, Yunnan, as well as the southern regions of Fujian, Guizhou, Xinjiang, and Taiwan, along with some areas in Sichuan province. Under the future climate scenarios, the distribution range of *A. odoratissima* will expand northward, but the core distribution area will still remain relatively unchanged. The research results can provide reference for the investigation, collection, and protection of natural resources of *A. odoratissima*.

## Figures and Tables

**Table 1:** TSS and ROC evaluations of ensemble model.

Variables	TSS	ROC
EMmean	0.894	0.972
EMmedian	0.896	0.967
EMca	0.897	0.979
EMwmean	0.895	0.973

## Data Availability

The datasets analyzed during the current study are available from the corresponding author upon reasonable request.

## Supplementary Materials

The following supporting information can be downloaded at: https://www.mdpi.com/article/10.3390/biology14020180/s1. Table S1: The occurrence data for modeling; Table S2: Information on the environmental variables used in this study.
