# Supplementary material for: Predicting the Potential Suitable Distribution of Albizia odoratissima (L. f.) Benth. Under Climate Change Based on the Biomod2 Model
Source: Biology (Basel). 2025 Feb 10;14(2):180. doi: 10.3390/biology14020180 (PMC11851378; doi:10.3390/biology14020180)
Supplement: Supplementary file 1 [file biology-14-00180-s001.zip › Table S2.pdf]

Table S2 Information on the environmental variables used in this study.

Type	Variables	Description	Unit
bioclimatic variables	bio1	Annual Mean Temperature	◦C
	**bio2**	**Mean Diurnal Range (Mean of monthly (max temp - min temp))**	**◦C**
	bio3	Isothermality (BIO2/BIO7) (×100)	-
	**bio4**	**Temperature Seasonality (standard deviation ×100)**	**◦C**
	bio5	Max Temperature of Warmest Month	◦C
	bio6	Min Temperature of Coldest Month	◦C
	bio7	Temperature Annual Range (BIO5-BIO6)	◦C
	**bio8**	**Mean Temperature of Wettest Quarter**	**◦C**
	bio9	Mean Temperature of Driest Quarter	◦C
	bio10	Mean Temperature of Warmest Quarter	◦C
	**bio11**	**Mean Temperature of Coldest Quarter**	**◦C**
	**bio12**	**Annual Precipitation**	**mm**
	bio13	Precipitation of Wettest Month	mm
	**bio14**	**Precipitation of Driest Month**	**mm**
	**bio15**	**Precipitation Seasonality (Coefficient of Variation)**	**1**
	bio16	Precipitation of Wettest Quarter	mm
	bio17	Precipitation of Driest Quarter	mm
	**bio18**	**Precipitation of Warmest Quarter**	**mm**
	bio19	Precipitation of Coldest Quarter	mm
topographic variables	**Altitude**	**Elevation**	**m**
	**Slope**	**Slope**	**-**
	**Aspect**	**Aspect**	**-**
