# Supplementary material for: Predicting the Potential Suitable Distribution of Albizia odoratissima (L. f.) Benth. Under Climate Change Based on the Biomod2 Model
Source: Biology (Basel). 2025 Feb 10;14(2):180. doi: 10.3390/biology14020180 (PMC11851378; doi:10.3390/biology14020180)
Supplement: Supplementary file 1 [file biology-14-00180-s001.zip › Table S1.pdf]

Table S1 The occurrence data for modeling.

| Species | longitude | latitude |
|---|---|---|
| Albizia odoratissima | 109.51 | 18.25 |
| Albizia odoratissima | 109.70 | 18.62 |
| Albizia odoratissima | 109.17 | 18.75 |
| Albizia odoratissima | 110.40 | 18.80 |
| Albizia odoratissima | 109.83 | 19.03 |
| Albizia odoratissima | 108.63 | 19.10 |
| Albizia odoratissima | 109.05 | 19.25 |
| Albizia odoratissima | 110.00 | 19.73 |
| Albizia odoratissima | 110.17 | 20.33 |
| Albizia odoratissima | 101.57 | 21.48 |
| Albizia odoratissima | 110.86 | 21.65 |
| Albizia odoratissima | 108.35 | 21.77 |
| Albizia odoratissima | 100.99 | 21.80 |
| Albizia odoratissima | 111.98 | 21.86 |
| Albizia odoratissima | 100.45 | 21.97 |
| Albizia odoratissima | 111.00 | 22.01 |
| Albizia odoratissima | 107.33 | 22.01 |
| Albizia odoratissima | 106.82 | 22.39 |
| Albizia odoratissima | 100.86 | 22.70 |
| Albizia odoratissima | 107.11 | 22.76 |
| Albizia odoratissima | 113.61 | 22.77 |
| Albizia odoratissima | 103.09 | 22.79 |
| Albizia odoratissima | 108.23 | 23.23 |
| Albizia odoratissima | 102.83 | 23.23 |
| Albizia odoratissima | 105.82 | 23.26 |
| Albizia odoratissima | 103.40 | 23.37 |
| Albizia odoratissima | 116.70 | 23.37 |
| Albizia odoratissima | 106.63 | 23.40 |
| Albizia odoratissima | 101.68 | 23.43 |
| Albizia odoratissima | 99.40 | 23.55 |
| Albizia odoratissima | 102.02 | 23.61 |
| Albizia odoratissima | 105.62 | 23.63 |
| Albizia odoratissima | 106.62 | 23.90 |
| Albizia odoratissima | 108.10 | 23.93 |
| Albizia odoratissima | 97.85 | 24.02 |
| Albizia odoratissima | 101.98 | 24.07 |
| Albizia odoratissima | 107.25 | 24.15 |
| Albizia odoratissima | 110.24 | 24.18 |
| Albizia odoratissima | 100.83 | 24.45 |
| Albizia odoratissima | 107.41 | 24.52 |
| Albizia odoratissima | 98.68 | 24.58 |
| Albizia odoratissima | 105.33 | 24.68 |

| | | |
|---|---|---|
| Albizia odoratissima | 101.63 | 24.70 |
| Albizia odoratissima | 97.93 | 24.71 |
| Albizia odoratissima | 113.27 | 24.78 |
| Albizia odoratissima | 102.40 | 24.81 |
| Albizia odoratissima | 105.84 | 24.93 |
| Albizia odoratissima | 98.81 | 24.94 |
| Albizia odoratissima | 105.47 | 25.12 |
| Albizia odoratissima | 110.20 | 25.23 |
| Albizia odoratissima | 106.75 | 25.43 |
| Albizia odoratissima | 102.47 | 25.55 |
| Albizia odoratissima | 110.67 | 25.62 |
| Albizia odoratissima | 101.88 | 25.70 |
| Albizia odoratissima | 99.95 | 26.11 |
| Albizia odoratissima | 107.80 | 26.20 |
| Albizia odoratissima | 103.30 | 26.42 |
| Albizia odoratissima | 100.18 | 26.57 |
| Albizia odoratissima | 101.71 | 26.58 |
| Albizia odoratissima | 102.25 | 26.67 |
| Albizia odoratissima | 104.72 | 27.13 |
| Albizia odoratissima | 102.27 | 27.90 |
| Albizia odoratissima | 108.40 | 28.00 |
| Albizia odoratissima | 104.52 | 28.43 |
| Albizia odoratissima | 103.95 | 28.60 |
